# More Fat, Less Bone? Flame Retardant May Deliver a One–Two Punch

**DOI:** 10.1289/ehp.122-A312

**Published:** 2014-11-01

**Authors:** Wendee Nicole

**Affiliations:** Wendee Nicole was awarded the inaugural Mongabay Prize for Environmental Reporting in 2013. She writes for *Discover*, *Scientific American*, *National Wildlife*, and other magazines.

Firemaster^®^ 550 (FM550) was introduced in 2003 as an alternative to the toxic, persistent flame retardant pentabromodiphenyl ether, for use in mattresses, couches, and other items containing polyurethane foam.^1^ FM550 contains a mixture of brominated phthalates and organophosphates. In 2013 a groundbreaking study found that pre- and postnatal exposure to FM550 was associated with increased anxiety, obesity, and early-onset puberty in rats, raising concern over the continued use of these chemicals.^2^ In this issue of *EHP*, a team of investigators report further evidence that components of FM550 may act as environmental obesogens, stimulating adipogenesis (fat formation) at the expense of bone health.^3^

**Figure:**
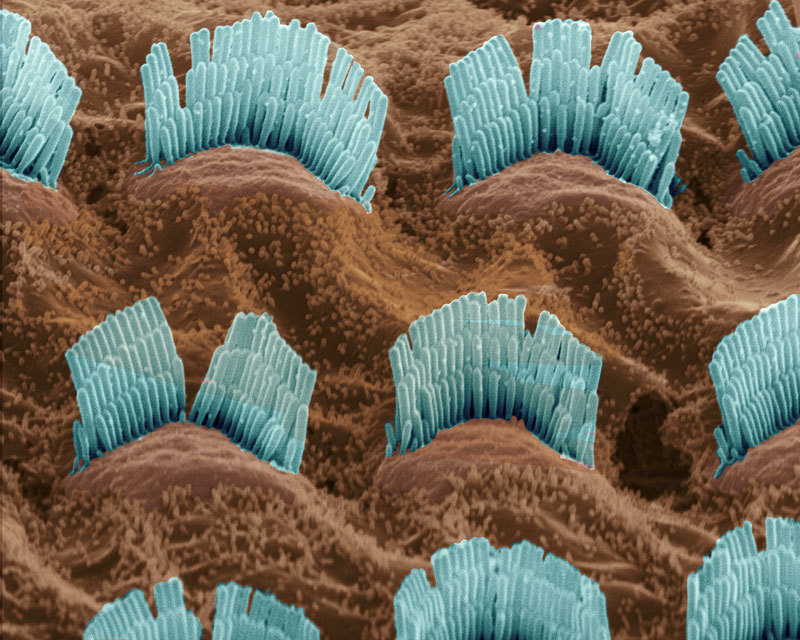
House dust can be a major source of exposure to flame retardants. Finger: © Gunnar Pippel/Shutterstock; TPP molecule: Pillai et al. (2014)^3^

Using computer modeling and receptor binding and activity assays, the authors of the current study found that the phosphate components of FM550 bound to and activated peroxisome proliferator–activated receptor γ, the master regulator of adipogenesis. The brominated components of FM550 did not. Using multipotent mesenchymal stromal cells from rats, they also showed that FM550 and its constituent triphenyl phosphate (TPP) stimulated formation of fat cells.^3^

When stem cells differentiate into fat cells, they do so at the expense of bone and cartilage formation.^4^ In this study, both FM550 and TPP suppressed osteogenesis in bone marrow cultures, with FM550 suppressing activity more than TPP alone.^3^ The authors hypothesize that environmental obesogens could be contributing to the growing prevalence of osteoporosis worldwide.^5^

House dust contains high levels of organophosphate flame retardants, and their metabolites are ubiquitous in human urine.^6^ The authors estimated that young children could ingest 120 μg/day TPP from indoor exposure to dust alone.^3^

“The effects shown in this paper occur at significantly higher exposures; thus, at present exposure levels there seems to be a margin of safety,” says Jerry Heindel, a health scientist administrator at the National Institute of Environmental Health Sciences, which supported the study in part. “However, if exposure increases due to increased use of FM550, the margin of safety could be eroded.”

According to Barbara Corkey, director of the Obesity Research Center at Boston University School of Medicine, the new study is important to the field “because FM550 is one of thousands of chemicals that have appeared in our environment since the obesity epidemic began. The important question this study raises is how many of the other thousands of compounds that have not been tested [may] also have a small effect on obesity.” Corkey was not involved in the research.

“This is the first study to provide evidence that an organophosphate-based flame retardant could contribute to bone loss,” says coauthor Jennifer Schlezinger, an associate professor of environmental health at Boston University School of Public Health. Although the idea that environmental chemicals could be contributing to the obesity epidemic is gaining ground,^4^ “few people think about what the consequences of environmental nuclear receptor ligands may be in the bone,” Schlezinger says.

Other research has found that the anti-diabetes drug rosiglitazone both increases the risk of bone fracture and acts as an obesogen,^7^ and tributyltin has been shown to induce adipogenesis and suppress osteogenesis.^8^ Arsenic and lead also may inhibit bone formation.^9^^,^^10^ “Each time I find that a chemical can suppress bone formation I become more intrigued and concerned with the idea that common environment contaminants are contributing to the onset or exacerbation of osteoporosis,” Schlezinger adds.

“This is a good study because it is comprehensive, asking the same question multiple times with different assays,” says Heindel. “These data match the physiology, as TPP … is structurally similar to tributyltin, which causes these same effects but at lower doses.”

The next steps will involve an expanded, improved risk assessment to the human population, Heindel says. “It is highly likely that similar effects will be seen in humans, as all these same pathways exist,” he says. “Indeed, tributyltin has been shown to affect human mesenchymal stem cells in the same manner as rodent stem cells used here.”^11^
